# Supplementary material for: Could the Inflation Reduction Act Maximum Fair Price Hurt Patients?
Source: J Health Econ Outcomes Res. 2024 Nov 27;11(2):154–60. doi: 10.36469/001c.125251 (PMC11612897; doi:10.36469/001c.125251)
Supplement: Online Supplementary Material [file jheor_2024_11_2_125251_253722.pdf]

Journal of Health Economics and Outcomes Research

Online Supplementary Material

Could the Inflation Reduction Act Maximum Fair Price Hurt Patients? *JHEOR*. 2024;11(2):??-??. doi:10.36469/jheor.2024.125251

Table S1: Calculated Copay Costs for Medicare Part D Apixaban and Rivaroxaban Prescriptions Managed by the 3 Largest PBMs (2023 $US)

Table S2: Differences in Midpoint Copay Costs If Apixaban and Rivaroxaban Moved to Tier 6 (2023 $US)

Figure S1: Relationship Between Copay Amounts and Treatment Abandonment

Figure S2: Present and Predicted Increases in Treatment Abandonment

This supplementary material has been provided by the authors to give readers additional information about their work.

This is an open-access article distributed under the terms of the Creative Commons Attribution 4.0 International License (CCBY-4.0). View this license's legal deed at http://creativecommons.org/licenses/by/4.0 and legal code at http://creativecommons.org/licenses/by/4.0/legalcode for more information.

Table S1. Calculated Copay Costs for Medicare Part D Apixaban and Rivaroxaban Prescriptions Managed by the 3 Largest PBMs (2023 $US)

Drug	PBM	Calculated Copay Costs (in Million 2023 $US)					
		2023			If All Moved to Tier 6		
		Min	Mid	Max	Min	Mid	Max
Apixaban (Eliquis)	CVS	347	532	723	460	705	958
	ORx	225	351	482	333	512	695
	ESI	181	276	374	195	301	408
	**Top 3 PBMs**	**753**	**1159**	**1579**	**988**	**1518**	**2061**
Rivaroxaban (Xarelto)	CVS	148	221	296	194	287	384
	ORx	83	127	172	130	192	257
	ESI	66	98	132	78	116	155
	**Top 3 PBMs**	**297**	**446**	**590**	**402**	**595**	**796**

The formulas used to derive these values are described in the Modeling section.
Abbreviations: CVS Caremark, CVS; Express Scripts International, ESI; ORx, Max, maximum; Min, minimum; Mid; midpoint; ORx, Optum Rx; PBM, pharmacy benefit manager.

Table S2. Differences in Midpoint Copay Costs If Apixaban and Rivaroxaban Moved to Tier 6 (2023 $US)

Drug	PBM	Calculated Copay Costs (in Million 2023 $US)		Difference (All Tier 6 vs 2023 Actual)
		2023	All Tier 6	
Apixaban (Eliquis)	CVS	532	705	173
	ORx	351	512	161
	ESI	276	301	25
	**Top 3 PBMs**	**1159**	**1518**	**359**
Rivaroxaban (Xarelto)	CVS	221	287	66
	ORx	127	192	65
	ESI	98	116	18
	**Top 3 PBMs**	**446**	**595**	**149**

The formulas used to derive these values are described in the Modeling section.
Abbreviations: CVS Caremark, CVS; Express Scripts International, ESI; ORx, Optum Rx; PBM, pharmacy benefit manager.

Figure S1. Relationship Between Copay Amounts and Treatment Abandonment

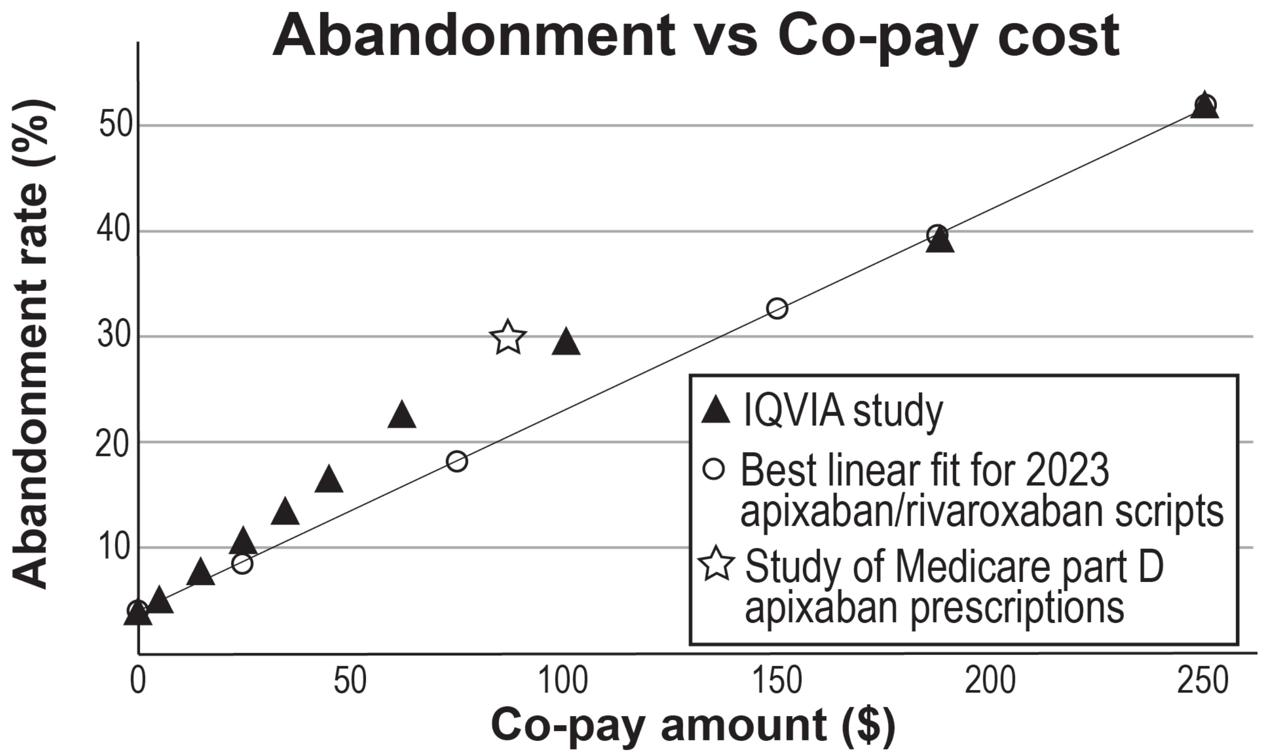

Figure S2. Present and Predicted Increases in Treatment Abandonment

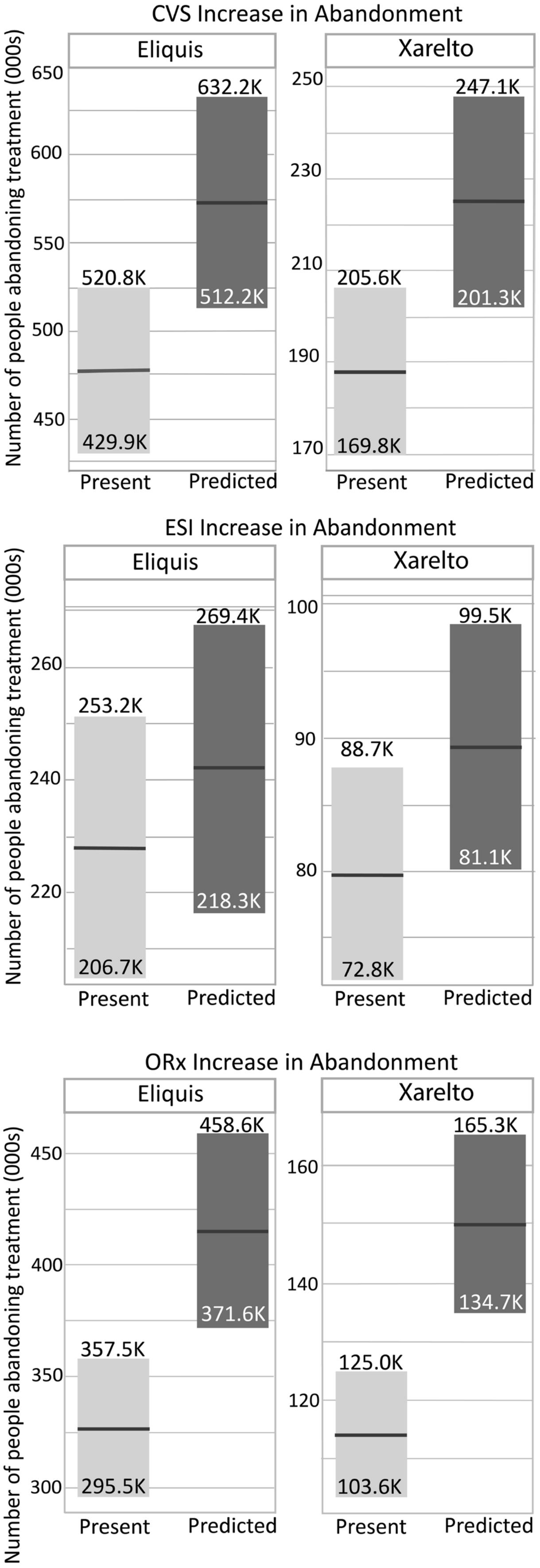

Abbreviations: CVS, CVS Caremark; ESI, Express Scripts International; ORx, Optum Rx.
