# Supplementary material for: Could the Inflation Reduction Act Maximum Fair Price Hurt Patients?
Source: J Health Econ Outcomes Res. 2024 Nov 27;11(2):154–60. doi: 10.36469/001c.125251 (PMC11612897; doi:10.36469/001c.125251)
Supplement: Article PDF [file jheor_2024_11_2_125251_255883.pdf]

Sydor AM, Rivera E, Popovian R. Could the Inflation Reduction Act maximum fair price hurt patients? *JHEOR*. **2024:11(2):154-160.** doi:10.36469/jheor.2024.125251

Journal of Health Economics and Outcomes Research

Brief Report

Could the Inflation Reduction Act Maximum Fair Price Hurt Patients?

Anne M. Sydor[1]*, Esteban Rivera[1], Robert Popovian[1,2]

[1]Global Healthy Living Foundation, Upper Nyack, New York, USA
[2]Pioneer Institute, Boston, Massachusetts, USA

ARTICLE INFORMATION

Article history:
Received September 4, 2024
Received in revised form October 28, 2024
Accepted October 29, 2024

Keywords: Inflation Reduction Act, Medicare Drug Price Negotiation Program, pharmacy benefit managers, maximum fair price, patients, out-of-pocket costs, formulary policy

*Corresponding author:
Email address: asydor@ghlf.org

➤ Supplementary Material

ABSTRACT

Background: The Inflation Reduction Act's Medicare Drug Price Negotiation Program allows the federal government to negotiate caps for select medications. These price caps may reduce revenue for the pharmacy benefit managers (PBMs) that negotiate the actual price paid for medicines in the U.S. To offset the resulting pressure on their profit margins, it is possible that PBMs would, in turn, increase patients' out-of-pocket costs for medicines with capped prices. The model presented here evaluates how increased out-of-pocket costs for the anticoagulants apixaban (Eliquis) and rivaroxaban (Xarelto) could impact patients financially and clinically.

Methods: Copay distributions for all 2023 prescription fills for apixaban and rivaroxaban managed by the 3 largest PBMs, CVS Caremark, Express Scripts International, and Optum Rx, were used to approximate current copay costs. Increased out-of-pocket costs were modeled as a shift of all apixaban and rivaroxaban prescriptions to the highest copay tier. The known linear relationship between copay costs and treatment abandonment was used to calculate the potential resulting increase in treatment abandonment. Known rates of morbidity and mortality due to abandoning anticoagulants were used to estimate resulting increases in morbidity and mortality.

Results: If the 3 largest PBMs all shifted costs onto patients by moving all apixaban and rivaroxaban prescriptions to the highest formulary tier, Tier 6, patients' copay amount would increase by $235 to $482 million for apixaban and $105 to $206 million for rivaroxaban. Such an increase could lead to 169 000 to 228 000 patients abandoning apixaban and 71 000 to 93 000 abandoning rivaroxaban. The resulting morbidity and mortality could include up to an additional 145 000 major cardiovascular events and up to 97 000 more deaths.

Conclusion: The Medicare Price Negotiation Program could impact patients negatively if it causes PBMs to increase patients' out-of-pocket costs for medicines. Policymakers should closely monitor changes in overall affordability, including all patient out-of-pocket expenditures, for medications in the program. Preemptive measures should be considered to ensure that the most vulnerable citizens are not placed in precarious situations, leading to poorer health outcomes.

INTRODUCTION

As with any complex U.S. federal legislation, the Inflation Reduction Act (IRA), signed into law on August 16, 2022, includes provisions with positive and negative consequences for diverse stakeholders.[1] Specific clauses affect health insurance premiums, patients' out-of-pocket costs, and wholesale medication prices that will impact patients, biopharmaceutical companies, the insurance industry, and policymakers differently, depending on their perspectives and roles within the healthcare system.

Eliminating out-of-pocket vaccination costs for seniors is an example with mostly positive benefits because costs will be reduced for patients, increasing access to these potentially life-saving interventions.[2,3] Other policy shifts, such as capping out-of-pocket costs and negotiating wholesale price limits may not be as beneficial for patients. For example, limiting out-of-pocket expenses to $2000 will benefit only 1 in 5 Medicare enrollees who currently pay more than that.[4] However, this cap could incentivize increasing costs to the upper limit for those patients who currently pay less. Pharmacy benefit management (PBM) companies may also increase burdensome utilization management techniques to offset their increased costs.[5-7]

This is an open-access article distributed under the terms of the Creative Commons Attribution 4.0 International License (CCBY-4.0). View this license's legal deed at http://creativecommons.org/licenses/by/4.0 and legal code at http://creativecommons.org/licenses/by/4.0/legalcode for more information.

The IRA authorized the Department of Health and Human Services to negotiate a "maximum fair price" (MFP) directly with manufacturers for some of the most highly used brand-name medications paid for by Medicare.[1] Many parties have raised concerns about this price setting.[8-11] An analysis from the Congressional Budget Office concluded this program would limit innovation, stall research and development, and likely reduce the number of new drug applications.[12]

At face value, lower wholesale medication prices appear beneficial to patients and harmful to biopharmaceutical manufacturers. However, this does not account for how medicines are purchased in the U.S., where the wholesale list price is rarely, if ever, the amount that a manufacturer receives for a medication. Almost all prescription medications are purchased under agreements negotiated by PBMs. Such agreements grant concessions (eg, fees, discounts, and rebates) to the insurer and PBMs but not to patients.[13-15] Notably, the net price paid for medicines is not publicly available, which makes it impossible to know how reduced wholesale prices affect the actual cost of medications for patients.[16] The negotiated net price paid by PBMs is based on the manufacturer's wholesale list price. Higher list prices create more room for negotiating concessions, whereas lowered, set prices will likely reduce concessions from manufacturers to the PBMs.[17,18] These dynamics, with IRA-mandated caps on out-of-pocket costs and premiums, will likely increase financial pressure on PBMs and insurers,[1] incentivizing them to shift costs elsewhere. Patients may be negatively impacted if PBMs and insurers shift greater costs to patients or restrict access to life-saving medicines.

PBMs manage patients' access to medications with formularies (ie, lists of medicines an insurer or PBM will cover) that exchange coverage of specific treatments for larger concessions from biopharmaceutical manufacturers,[18] which incentivizes covering higher-priced medications that drive larger concessions. These misaligned incentives have led to hundreds of medications being excluded from formularies or placed on higher tiers where patient out-of-pocket costs are more significant, which can harm patients medically and financially.[19-27] There is a known linear relationship between patients' out-of-pocket costs for a medication and the number of patients who abandon, or stop using that medication.[22-27] As out-of-pocket costs rise, the use of effective treatments declines, and this disproportionately affects people from marginalized communities.[26] Sometimes patients who stop using a treatment may find a cheaper alternative, but more than half the time this is not medically equivalent and may be less effective for a given individual patient.[18-19] In a study of Medicare Part D beneficiaries subject to a mean $81 copay increase for the direct oral anticoagulant (DOAC) apixaban between 2016 and 2017, 30% stopped using any prescription anticoagulant.[28]

To evaluate the potential effects of shifting costs to patients due to the MFP provisions of the IRA, we examined current out-of-pocket costs for 2 DOACs, apixaban (Eliquis) and rivaroxaban (Xarelto)–among the first 10 drugs selected for the Medicare Drug Price Negotiation Program–and how these would change if these drugs were moved to a higher formulary tier. We used the out-of-pocket cost changes from a formulary shift as a proxy for a shift of costs onto patients because data showing actual, real-world costs for patients by formulary tier is available vs other means of cost shifting (eg, increased copays without changing tier structure). Lastly, we evaluated the potential clinical impact of higher costs increasing the number of patients who abandon treatment and the resulting adverse outcomes.

METHODS

We evaluated the potential economic impact of the 3 largest PBMs—CVS Caremark (CVS), Express Scripts International (ESI), and Optum Rx (ORx)—which negotiate prices for over 80% of all prescriptions in the U.S.,[21] on patients' increased out-of-pocket costs for apixaban and rivaroxaban. Tier switching was used as a surrogate to estimate changes to patients' out-of-pocket costs. We modeled how moving apixaban or rivaroxaban to Tier 6 from Tier 3 in the 3 largest PBM formularies would affect (1) patients' annual out-of-pocket costs for these medications, (2) the number of people who would likely stop taking their prescribed apixaban or rivaroxaban, and (3) the resulting morbidity or all-cause mortality. This evaluation presents a theoretical model of what could happen and does not compare actual before and after data. Thus, no data comparisons were made, and no hypotheses were tested. As such, a statistical analysis was neither possible nor appropriate.

Data Sources, Structure, and Key Variables

Pfizer Inc. supplied unpublished data on the number of total Medicare Part D prescriptions by formulary tier and copay cost ranges in 2023 for all apixaban and rivaroxaban prescription fills managed by CVS, ORx, and ESI. These data can be purchased from third-party aggregators of medical claims and prescription fill data in the U.S. This is a limited data set in that it provides only the number of prescription fills by month and copay within a formulary tier with no demographic information (eg, age, ethnicity, gender, race, socioeconomic status of the patients filling prescriptions). To our knowledge, this is the only available data on what patients pay for rivaroxaban and apixaban prescriptions.

Published IQVIA data, representative of the U.S. population, was used to estimate how out-of-pocket expenditures affect abandonment rates.[22] Although a lack of patient-level demographic data also limits these, they were used because they are from a study of prescription claims in a representative sample across more than 25 drug classes.[22,23] Other studies, showing a similar linear relationship have demographic data but are not as generalizable because they evaluate only a single medication or medication class.[24-27]

Multiple studies show that discontinuing an oral anticoagulant increases the risk of all-cause mortality, ischemic stroke, and systemic embolism.[29-37] In 2 real-world cohorts followed for 6.5 and 1.4 years, the hazard ratio (HR) for all-cause mortality after discontinuing anticoagulants was 1.3 and 1.6, respectively.[29,30] We used the lower of these as the more conservative estimate in our model. Those followed for 6.5 years had an HR of 1.45 for major cardiovascular events.[29] Those followed for 1.4 years had HR 1.85 for myocardial infarction and 2.2 for ischemic stroke.[30] Other studies of anticoagulant discontinuation showed increased HRs for major cardiovascular events or ischemic stroke ranging from 1.74 to 3.9.[31-37] We used the 1.45 HR for major cardiovascular events after discontinuation because it came from the study with the longest follow-up period and provided the most conservative estimate.[29]

Model Assumptions

Rather than theorizing what PBM behavior might be, we used actual data reflecting prior PBM behavior (ie, the copay range structure in 2023) as a proxy for increased out-of-pocket costs. Although PBMs have other ways to raise patients' out-of-pocket costs (eg, higher copays within formulary tiers), we found no publicly available data on such patterns to use in this modeling study. Because the data available to us showed all Medicare Part D formularies used either formulary Tier 3 or Tier 6 copays, we used the range of copays within those tiers only when modeling a potential increase in out-of-pocket costs for CVS and ORx Tier 3 prescriptions. The Tier 6 copay structure for ESI was used to calculate the minimum, maximum, and midpoint of a potential shift from Tier 3 to Tier 6 for all apixaban and rivaroxaban prescriptions, which provides an internal sensitivity analysis. The midpoint of the

range was used to calculate total costs (eg, $75 was used for the $50-$100 range). All out-of-pocket cost increases depended on the difference in structure between Tier 3 and Tier 6 by ESI in 2023.

To model potential abandonment and increases in morbidity and mortality, we assumed a 90-day prescription refill across all data and thus divided total annual prescriptions (Pfizer unpublished data) by 4 to estimate the number of individuals who took either apixaban or rivaroxaban in 2023. It is possible that many prescriptions were for less than 90 days; however, since a 90-day prescription is the longest available, we used this conservative estimate to reduce the risk of counting individuals more than once (ie, assuming 30-day prescriptions would substantially increase the estimated number of patients affected).

Multiple studies have shown a linear relationship between the abandonment of treatment and copay costs.[22-27] Data from the most recent study with the largest, most generalizable data (ie, millions of patients and more than 25 drug classes) was used to estimate how many people would abandon treatment within each out-of-pocket dollar amount.[22]

A 45% and 30% increase, respectively, in the likelihood of major cardiovascular events and all-cause mortality in people who abandon anticoagulant treatment was used to estimate resulting increases in morbidity and mortality.[29]

Modeling

Out-of-pocket costs depended on 2023 total prescription fills (TRx), the percentage of total prescriptions within a given tier (%TRx), and the percentage breakdown of each copay range within each tier (%TTRx). Total prescription costs for each tier were calculated as the sum of the cost per range across all 5 copay amounts within a tier for all tiers, with cost per range calculated as:

$$\text{Cost per Range (\$)} = \text{Copay(\$)} * (\text{TRx Within Tier} * \%\text{TRx Within Range})$$

Current costs were calculated based on copay structures for each PBM in 2023. Potential costs were calculated utilizing the ESI Tier 6 copay structure. The difference in the copay range structure of Tier 3 and Tier 6 determined the cost differences per range. The total change in out-of-pocket costs was calculated as the difference between the potential costs if apixaban and rivaroxaban were moved to formulary Tier 6 and the 2023 copay costs as calculated above.

Using the relationship between out-of-pocket-costs and abandonment,[22] a simple linear regression of

$$\text{Y (Abandonment Rate)} = \text{mX (Copay)} + \text{b (Intercept When Cost = 0)}$$

was used to estimate resulting changes in abandonment rates after dividing total prescriptions by 4 assuming that with 90-day refills, every 4 prescriptions are equivalent to 1 person using apixaban or rivaroxaban during 2023. Potential changes in abandonment rates were estimated based on the midpoint and maximum of each copay range to approximate the range of people likely to abandon treatment because of increased out-of-pocket costs. These rate estimates were multiplied by total prescriptions within each copay range to calculate total abandonment for each range as:

$$\text{Total Abandonment Per Range} = \text{Predicted Abandonment} * \text{TRx Within Copay Range}$$

The increase in the number of people who would abandon apixaban or rivaroxaban treatment was calculated as the difference in the total abandonments if all prescriptions were moved to Tier 6 and the likely abandonment rate in 2023. Increased morbidity and mortality were calculated as 45% and 30%, respectively, of the increased abandonments likely to occur if all apixaban and rivaroxaban prescriptions were moved to Tier 6.[29]

RESULTS

Potential Cost Increases

Table 1 shows there were 23.8 million prescriptions for apixaban or rivaroxaban covered by Medicare Part D in 2023, and **Table 2** shows patients' copay amounts, which were only available for ranges of copay values. Notably, the proportion of patients with different copay ranges varied from 0.5% to 24.2%. Only ESI used Tier 6 for Medicare Part D apixaban and rivaroxaban prescriptions, and this copay distribution was used to estimate changed out-of-pocket costs if all 3 PBMs moved prescriptions for apixaban and rivaroxaban to a higher tier (**Table 3**).

Using the cost-range midpoints, the cumulative copay increases from all 3 of the largest PBMs shifting Medicare Part D prescriptions to Tier 6 (**Supplemental Table S2**) would be $359 million (CVS: $173 million, ORx: $160 million, and ESI: $25 million) for apixaban and $149 million (CVS: $66 million, ORx $65 million, and ESI: $18 million) for rivaroxaban. Differences between PBMs reflect differences in the number of prescriptions filled by each (**Table 1**) and the varied distributions of patients within copay ranges for each PBM (**Table 2**).

Abandonment, Mortality, and Morbidity

As described in the methods, we conservatively estimated the number of patients currently taking apixaban or rivaroxaban by assuming that all patients with Medicare Part D were using 90-day prescription fills for these medications. Under that assumption, we estimated that 5 951 405 patients with Medicare Part D coverage were taking one of these medications and paying a Tier 3 or Tier 6 formulary copay. Data from IQVIA shows a linear relationship between copay amounts and the proportion of patients who stop using a medication,[25] which we used to calculate the best linear fit slope and coefficient (**Supplemental Figure S1**). Applying that linear coefficient to the number of patients who would be affected by out-of-pocket cost increases because of a formulary tier shift, we calculated that between 169 000 and 228 000 (apixaban) or 71 000 to 93 000 (rivaroxaban), would abandon treatment (**Table 3**) because of the increased financial burden of treatment. Because not all patients pay the same copays, even when the medication

Table 1. Number of Prescriptions for Apixaban and Rivaroxaban Managed by the 3 Largest PBMs as Formulary Tier 3 and Tier 6 in 2023[a]

	CVS Tier 3	ORx Tier 3	ESI Tier 3	ESI Tier 6[b]
Apixaban (Eliquis)	8 125 970	5 895 443	3 463 353	126 929
Rivaroxban (Xarelto)	2 968 237	1 985 963	1 195 899	43 829

[a]Data provided by Pfizer, Inc.

[b]Only ESI currently uses Tier 6 for apixaban and rivaroxaban prescriptions.

Abbreviations: CVS Caremark, CVS; Express Scripts International, ESI; ORx, Optum Rx; PBM, pharmacy benefit manager.

Table 2. Proportion (%) of Prescriptions for Apixaban and Rivaroxaban by Tier and Copay Range in the 3 Largest PBMs[a]

Drug/Copay Range	CVS Tier 3	ORx Tier 3	ESI Tier 3	ESI Tier 6[b]
Apixaban (Eliquis)				
$0	25.3	27.3	24.8	18.3
>$0-50	29.4	26.3	30.7	30.7
$50-100	25.9	30.6	6.6	8.4
$100-200	9.9	9.3	27.6	33.5
>$200	9.5	6.5	10.3	9.0
Rivaroxaban (Xarelto)				
$0	23.4	28.6	26.6	17.0
>$0-50	25.4	22.4	28.3	27.6
$50-100	26.3	29.7	6.3	8.7
$100-200	13.8	12.2	26.8	33.4
>$200	11.1	7.1	12.0	13.3

[a]Data provided by Pfizer, Inc.
[b]Only ESI currently uses Tier 6 for apixaban and rivaroxaban prescriptions.
Abbreviations: CVS Caremark, CVS; Express Scripts International, ESI; ORx, Optum Rx; PBM, pharmacy benefit manager.

Table 3. Increased Number of Patients Who Would Abandon Treatment With Apixaban and Rivaroxaban If Moved to Formulary Tier 6

	Eliquis		Xarelto	
	**Minimum**	**Maximum**	**Minimum**	**Maximum**
CVS	82 000	111 000	32 000	42 000
ORx	76 000	101 000	31 000	40 000
ESI	11 000	16 000	8 000	11 000
**3 largest PBMs**	**169 000**	**228 000**	**71 000**	**93 000**

The number of patients likely to abandon treatment was calculated by applying a linear equation of the known relationship between patient copay costs and treatment abandonment, as detailed in **Supplementary Figures S1 and S2**.
Abbreviations: CVS, CVS Caremark; ESI, Express Scripts International; ORx, Optum Rx; PBM, pharmacy benefit manager.

is on the same tier in different formularies, we calculated the minimum and maximum cost increases in **Table 3**.

Using the 45% increased risk of major cardiovascular events and the 30% increased risk of all-cause mortality after abandoning anticoagulants,[21] an estimated 76 000 to 103 000 more major cardiovascular events would result among those patients who abandoned apixaban and 32 000 to 42 000 events for patients who abandoned rivaroxaban. Among those abandoning apixaban and rivaroxaban, increases in all-cause mortality are projected at 51 000 to 69 000 and 21 000 to 28 000, respectively.

DISCUSSION

The PBM contracting model in the U.S. incentivizes higher-rebated medicines, as PBMs retain much of the rebates collected as profit. Higher-priced medicines are preferred since fees are based on the retail price of the covered drug. Notably, fees collected by PBMs from manufacturers, such as assessments for specialty drug dispensing through PBM-owned pharmacies, are often undisclosed and not shared as savings for sponsors or patients.

As the federal government uses IRA to suppress wholesale prices of designated medicines, PBMs' profits are expected to decrease, even if manufacturers are forced to provide additional concessions. To recoup lost profits, PBMs may increase out-of-pocket costs for seniors, reduce sharing rebate concessions with plan sponsors, increase utilization management, or reduce pharmacy reimbursement. Although these policies may decrease cost for the Medicare program and for some patients who currently have out-of-pocket costs over $2000 per year,[4] our model suggests there is also potential negative impact on individual patients who may face increased cost-sharing requirements. Such out-of-pocket increases for patients affect their prescription fill behaviors, increase treatment abandonment, and can cause substantial increases in morbidity and mortality.

Data Limitations Show a Need for Increased Transparency

As with any modeling analysis, the projections reported here are limited by the data available. Publicly available, transparent, and detailed data on what patients pay for the medicines initially included in the MFP program are extremely limited. Neither PBMs, health insurance companies, nor biopharmaceutical manufacturers disclose the actual net prices paid for medications. PBMs may not even report to the plan sponsor what price was paid for a medication. Some data is available to third-party organizations that collect and sell medical claims data. However, these are typically aggregate data that do not include patient characteristics other than insurance type. This highlights a great need for more transparency about the actual prices paid for medicines in the U.S., without which all economic and cost-benefit analyses and models are limited. For this modeling study, we obtained actual copays paid by proportions of patients whose prescriptions were covered by Medicare Part D benefits, allowing us to construct a model based on actual copay distributions rather than theorizing what PBMs might do.

Data on copay amounts were available only as the proportions of filled prescriptions that fell within a range of copays (varying by increments of $50 or $100) for the current tiers for apixaban and rivaroxaban (Tier 3 and Tier 6). As a result, we could only calculate potential ranges of copay increases that would occur if people's prescriptions moved from Tier 3 to Tier 6. Similarly, because abandonment rates are dependent on copay amounts,[22-27] we could only calculate ranges for the number of people who would abandon treatment with such a formulary shift and the number with increased morbidity and mortality as a result of abandonment.

It must be acknowledged that raising copays by shifting medications from one formulary tier to another, which the available data allowed us to model, is only one of many ways that costs can be shifted to patients. Other tactics PBMs have used to shift costs to patients include spread pricing, increased coinsurance amounts, selective tier switching that affects some but not all patients, or imposing step therapy or prior authorization requirements. It is also possible that PBMs and insurers could increase copay amounts without changing formulary tiers, considering the broad range of copay costs within a tier that we report here. Data on such practices, however, are not available. Thus, instead of theorizing about potential policy changes, we created a model from actual PBM data, using a shift from one copay tier to another as a proxy for increased patient out-of-pocket costs, regardless of how such increases are achieved.

Another limitation of these data is that, although it includes all Medicare Part D prescriptions for rivaroxaban and apixaban in 2023, it does not include data on the characteristics of patients paying different copay amounts (eg, social determinants of health (eg, race, ethnicity, rural vs urban locations, or socioeconomic status). Because studies of the relationship between copay costs and treatment discontinuation for individual therapy classes show that negative social determinants of health can also increase treatment abandonment rates,[26] having such data would allow a more accurate understanding of this relationship. Such analysis could help elucidate any nonlinearity of this relationship. Because those data are unavailable, we used the known linear relationship across a representative sample of the US population and more than 25 medication classes as our best estimate. Supporting this choice, a study of apixaban discontinuation by Medicare Part D beneficiaries after a copay increase of $81 is consistent with the values in the linear relationship we used for estimation (**Supplemental Figure S1**).[28]

Because the paucity of available data sources limits the ability to perform sensitivity analyses for the presented model, we present all results generated by the model as a range from minimum to maximum with the median effect shown. Although other scenarios could be modeled, these would be based on guesses about PBM practices rather than historical data and thus would not provide adequate comparisons for sensitivity analysis.

In addition, only the total number of prescriptions filled by Medicare Part D enrollees was available. These fills had to be assumed as 90-day fills to roughly estimate the number of people taking the medications as one-fourth of all fills and avoid overcounting the number of people affected.

Potential for Harm to Individual Patients and Public Health

The data analyzed show that the 3 major PBMs—CVS, ORx, and ESI—do not follow a uniform benefit structure for patients' out-of-pocket costs. CVS and ORx place rivaroxaban and apixaban on Tier 3, with differing percentages of patients at each copay level within this tier. Consequently, patients requiring anticoagulant treatment may be better off with CVS and ORx plans. However, this also means that CVS and ORx have more room to increase out-of-pocket costs for seniors.

Past research demonstrates the impact of formulary exclusions on patients.[18-21] The analysis presented here suggests shifting out-of-pocket costs onto patients, which could occur as a consequence of the IRA MFP, may have a similar detrimental effect. Policies that move patients to higher out-of-pocket spending will not only strain seniors' finances but also force some to abandon treatments, leading to more severe health consequences, including increased morbidity and mortality as described.

The proportions of patients likely to discontinue rivaroxaban or apixaban with increased copay costs are known, as are some of the consequences of such discontinuation. Other adverse effects might include increased healthcare utilization, disability, and lost quality of life, although data on how discontinuing apixaban or rivaroxaban affects those needs to be generated. Additionally, it is well-established that not all patients discontinue treatment as copay costs rise. Some will continue on the medication at increased costs with negative consequences for their financial well-being. Others will switch to a cheaper medication that may be less effective, as is the case for rivaroxaban vs the generic anticoagulant warfarin, or may have a higher likelihood of adverse events (ie, side effects) for that individual. Lower efficacy and higher rates of adverse events are also likely to harm patients, public health, and the economy through higher healthcare costs. However, it is not possible to model these consequences because the proportion of patients who continue on the same more expensive medication vs switch to a cheaper medication after copay and other out-of-pocket cost increases is not known.[14]

Policy Recommendations

Nuances in benefit design, including the broad range of copay amounts paid by different proportions of patients for medications on the same formulary tier and effects on specific demographic groups, are critical for the Center for Medicaid and Medicare Services (CMS) to consider during price-setting efforts and follow-up monitoring. CMS must consider these consequences when monitoring health plans and scrutinizing the Medicare Part D benefit designs submitted by PBMs for such potential scenarios.

In this analysis, we used changes in medication tiers as a surrogate to examine potential increased out-of-pocket costs for seniors. However, focusing solely on medication tiers is inadequate. CMS should instead concentrate on the overall distribution of patient out-of-pocket expenditures for the anticoagulant class, regardless of the medication tier. The 3 PBMs might not shift patients to higher tiers but could increase out-of-pocket costs within the existing tiers. Policymakers should consider whether there is a need to better support seniors who cannot afford their medications. This could include making Medicare beneficiaries eligible for copay assistance programs, which they are not allowed to participate in now. Increased subsidies for patients who cannot afford their medications could also be considered. It is important to note, however, that these programs reduce biopharmaceutical company revenue, leading to reduced rebates that, in turn, encourage PBMs to shift even more costs to patients. Detailed analyses of all of these factors are needed when considering any policy change.

Among the most effective policy changes that could be made would be requiring transparent reporting by PBMs of all rebates, fees, and patient out-of-pocket costs by type (coinsurance, copay, and other) and patient demographics. Such granular data is necessary to do a complete cost-benefit analysis of the IRA that should include not only reduced costs to Medicare as a program but also cost changes for individual patients. In turn, this could allow analyses to incorporate a more refined assessment of treatment abandonment, increased morbidity and mortality, direct and indirect healthcare costs, and impacts on disability and quality of life.

Recommendations for Future Research

Future research should evaluate formulary patient out-of-pocket requirements and the distribution of patients across tiers for rivaroxaban and apixaban following the implementation of IRA price-setting requirements. Furthermore, CMS should assess each patient's needs individually, as PBMs may shift out-of-pocket costs to other commonly used generic medications. This modeling study is essential because it shows that negative effects are possible and should be monitored. Additionally, prospective studies of how patients would respond to

increased out-of-pocket costs for medications affected by MFPs should be conducted and could include patient surveys and discrete choice experiments. As MFPs go into effect, it will be necessary to prospectively evaluate how these affect patients' out-of-pocket costs, treatment abandonment, nonmedical switching of treatments, and related morbidity, mortality, disability, and healthcare costs.

CONCLUSION

Implementing IRA will offer specific benefits to seniors, such as lower out-of-pocket costs for seniors who hit the $2000 cap. However, not all outcomes will be favorable, particularly for patients relying on future cures and affordable access to treatments for chronic diseases. As the IRA exerts pressure on PBM's profits, it may trigger policy shifts that make it harder for patients currently stable on therapy to afford their medicines. To maintain profits, PBMs may shift costs to seniors through higher out-of-pocket requirements for both price-controlled drugs and other medications frequently used to manage chronic conditions. Such policies could have devastating effects on patients who depend on these treatments.

CMS and policymakers must closely monitor changes in overall affordability and take preemptive measures to ensure that the most vulnerable citizens are not placed in precarious situations leading to poorer health outcomes. This will require transparency from PBMs regarding rebates, fees, copays, coinsurance, and other patient out-of-pocket costs. Future research should focus on evaluating the impact of formulary design changes on patient out-of-pocket costs and adherence, especially under the new pricing dynamics introduced by the IRA. By proactively addressing these challenges, we can better safeguard the health and well-being of seniors.

REFERENCES

1. Inflation Reduction Act of 2022. HR, 5376, 117th Congress. Introduced 2021. Passed 2022.
2. Sayed BA, Feyman Y, King KL, et al. *Inflation Reduction Act Research Series: Medicare Part D Enrollee Vaccine Use After Elimination of Cost Sharing for Recommended Vaccines in 2023.* Office of the Assistant Secretary for Planning and Evaluation, US Department of Health and Human Services; 2024. https://aspe.hhs.gov/reports/ira-elimination-vaccine-cost-sharing-2023
3. Benjamin RM. The national prevention strategy: shifting the nation's health-care system. *Public Health Rep*. 2011;126(6):774-776. doi:10.1177/003335491112600602
4. Holtz-Eakin D. *The 10-percent Solution: Who Gets IRA Drug Price Savings?* American Action Forum; 2023. https://www.americanactionforum.org/research/the-10-percent-solution-who-gets-ira-drug-price-savings/
5. Cubanski J, Damico A, Neuman T. *How Medicare's New Drug Price Negotiation Program Could Expand Access to Selected Drugs.* Kaiser Family Foundation; 2023. https://www.kff.org/medicare/issue-brief/how-medicares-new-drug-price-negotiation-program-could-expand-access-to-selected-drugs/
6. Brantley K, Stengel K, Donthi S, Parekh S, Feuer A. *IRA Reforms Will Impact Patient Adherence and Affordability.* Avalere; 2023. https://avalere.com/insights/ira-reforms-will-impact-patient-adherence-and-affordability
7. Chandra A, Ippolito B. What does the Inflation Reduction Act mean for patients and physicians? *NEJM Catal Innov Care Deliv*. 2023;4(10)doi:10.1056/CAT.23.0138
8. Arnold LM, Crofford LJ, Mease PJ, et al. Patient perspectives on the impact of fibromyalgia. *Patient Educ Couns*. 2008;73(1):114-20. doi:10.1016/j.pec.2008.06.005
9. Philipson TJ, Durie T. *Issue Brief: The Impact of HR 5376 on Biopharmaceutical Innovation and Patient Health.* University of Chicago; 2021. https://ecchc.economics.uchicago.edu/files/2021/08/Issue-Brief-Drug-Pricing-in-HR-5376-11.30.pdf
10. Gassull D, Bowen H, Schulthess D. IRA's impact on the U.S. biopharma ecosystem. *Vital Transformation;* 2023. https://vitaltransformation.com/2023/05/iras-impact-on-the-us-biopharma-ecosystem/
11. Grogan J. The Inflation Reduction Act is already killing potential cures. *Wall Street Journal.* Nov. 3, 2022. https://www.wsj.com/articles/the-inflation-reduction-act-killing-potential-cures-pharmaceutical-companies-treatment-patients-drugs-prescriptions-ira-manufacturers-11667508291
12. *Estimated Budgetary Effects of Public Law 117-169, to Provide for Reconciliation Pursuant to Title II of S. Con. Res. 14.* Congressional Budget Office. 2022. https://www.cbo.gov/system/files/2022-09/PL117-169_9-7-22.pdf
13. Purchaser Business Group on Health. *The Hidden Cost of PBMS in the Health Care Industry.* 2023. https://www.pbgh.org/the-hidden-cost-of-pbms-in-the-health-care-industry/
14. Texas Department of Health Insurance. *Life, Health, and Accident Insurance Reports, Prescription Drug Cost Transparency, Pharmacy Benefit Managers*.
15. Fein AJ. Texas shows us where PBMs' rebates go. *Drug Channels;* 2022. https://www.drugchannels.net/2022/08/texas-shows-us-where-pbms-rebates-go.html
16. Pannier A. *TransparencyRx and the Mission to Fix Broken Drug Pricing*. SmithRx; 2023. https://www.smithrx.com/blog/transparencyrx-and-the-mission-to-fix-broken-drug-pricing
17. Feldman WB, Rome BN. The rise and fall of the insulin pricing bubble. *JAMA Netw Open.* 2023;6(6):e2318074. doi:10.1001/jamanetworkopen.2023.18074
18. National Association of Insurance Commissioners. *A Guide to Understanding Pharmacy Benefit Manager and Associated Stakeholder Regulation.* 2023. https://content.naic.org/sites/default/files/committee_related_documents/PBM%2520White%2520Paper%2520Draft%2520Adopted%2520B%2520Committee%252011-2-23_0.pdf
19. Crea S, Sydor AM, Popovian R. Analysis of drug formulary exclusions from the patient's perspective: 2023 update. *Health Sci J.* 2023;17(8). doi:10.36648/1791-809X.16.S8.002
20. Sydor AM, Bergin E, Kay J, Stone E, Popovian R. Modeling the effects of formulary exclusions: how many patients could be affected by a specific exclusion? *J Health Econ Outcomes Res.* 2024;11(1):86-93. doi:10.36469/001c.94544
21. Fein AJ. The top pharmacy benefit managers of 2023: market share and trends for the biggest companies—and what's ahead. *Drug Channels;* 2024. https://www.drugchannels.net/2024/04/the-top-pharmacy-benefit-managers-of.html
22. *The Use of Medicines in the U.S. 2024: Usage and Spending Trends and Outlook to 2028.* IQVIA; 2024. https://www.iqvia.com/insights/the-iqvia-institute/reports-and-publications/reports/the-use-of-medicines-in-the-us-2024
23. Devane K, Harris K, Kelly K. *Patient Affordability Part Two: Implications for Patient Behavior & Therapy Consumption.* IQVIA. 2018. https://www.iqvia.com/locations/united-states/library/case-studies/patient-affordability-part-two

24. Klebanoff MJ, Li P, Lin JK, Doshi JA. Formulary coverage of brand-name adalimumab and biosimilars across Medicare Part D plans. *JAMA*. 2024;332(1):74-76. doi:10.1001/jama.2024.8917
25. Doshi JA, Li P, Puckett J, Pettit A, Parmacek MS, and Rader D. Abstract 285: Association of patient out-of-pocket costs and financial assistance with prescription abandonment: the case of PCSK9 inhibitors. *Circ Cardiovasc Qual Outcomes.* 2020;13(suppl 1). doi:10.1161/hcq.13.suppl_1.285
26. Doshi JA, Li P, Huo H, Pettit AR, Armstrong KA. Association of patient out-of-pocket costs with prescription abandonment and delay in fills of novel oral anticancer agents. *J Clin Oncol.* 2018;36(5):476-482. doi:10.1200/JCO.2017.74.5091
27. Dean LT, Nunn AS, Chang HY, et al. Estimating the impact of out-of-pocket cost changes on abandonment of HIV pre-exposure prophylaxis. *Health Aff (Millwood).* 2024;43(1):36-45. doi:10.1377/hlthaff.2023.00808
28. Deitelzweig S, Terasawa E, Atreja N, et al. Payer formulary tier increases of apixaban: how patients respond and potential implications. *Curr Med Res Opin.* 2023;39(8):1093-1101. doi:10.1080/03007995.2023.2232636
29. Rivera-Caravaca JM, Roldán V, Esteve-Pastor MA, et al. Cessation of oral anticoagulation is an important risk factor for stroke and mortality in atrial fibrillation patients. *Thromb Haemost.* 2017;117(7):1448-1454. doi:10.1160/TH16-12-0961
30. Cools F, Johnson D, Camm AJ, et al. Risks associated with discontinuation of oral anticoagulation in newly diagnosed patients with atrial fibrillation: Results from the GARFIELD-AF Registry. *J Thromb Haemost.* 2021;19(9):2322-2334. doi:10.1111/jth.15415
31. Gallego P, Roldan V, Marín F, et al. Cessation of oral anticoagulation in relation to mortality and the risk of thrombotic events in patients with atrial fibrillation. *Thromb Haemost.* 2013;110(6):1189-1198. doi:10.1160/TH13-07-0556
32. Dagenais GR, Dyal L, Bosch JJ, et al. Cardiovascular consequences of discontinuing low-dose rivaroxaban in people with chronic coronary or peripheral artery disease. *Heart.* 2021;107(14):1130-1137. doi:10.1136/heartjnl-2020-318758
33. García Rodríguez LA, Cea Soriano L, Munk Hald S, et al. Discontinuation of oral anticoagulation in atrial fibrillation and risk of ischaemic stroke. *Heart.* Published online December 11, 2020. doi:10.1136/heartjnl-2020-317887
34. Martinez C, Wallenhorst C, Rietbrock S, Freedman B. Ischemic stroke and transient ischemic attack risk following vitamin K antagonist cessation in newly diagnosed atrial fibrillation: a cohort study. *J Am Heart Assoc.* 2020;9(2):e014376. doi:10.1161/JAHA.119.014376
35. Giehm-Reese M, Johansen MN, Kronborg MB, et al. Discontinuation of oral anticoagulation and risk of stroke and death after ablation for typical atrial flutter: a nation-wide Danish cohort study. *Int J Cardiol.* 2021;333:110-116. doi:10.1016/j.ijcard.2021.02.057
36. Mulholland RJ, Manca F, Ciminata G, et al. Evaluating the effect of inequalities in oral anti-coagulant prescribing on outcomes in people with atrial fibrillation. *Eur Heart J Open.* 2024;4(2):oeae016. doi:10.1093/ehjopen/oeae016
37. Carnicelli AP, Al-Khatib SM, Xavier D, et al. Premature permanent discontinuation of apixaban or warfarin in patients with atrial fibrillation. *Heart.* 2021;107(9):713-720. doi:10.1136/heartjnl-2020-317229
